# A sibling study of whether maternal exposure to different types of natural space is related to birthweight

**DOI:** 10.1093/ije/dyx258

**Published:** 2017-12-13

**Authors:** Elizabeth A Richardson, Niamh K Shortt, Richard Mitchell, Jamie Pearce

**Affiliations:** 1 National Health Service Health Scotland, Edinburgh, UK; 2 Centre for Research on Environment, Society and Health (CRESH), University of Edinburgh, Edinburgh, UK and; 3 Centre for Research on Environment, Society and Health (CRESH), University of Glasgow, Glasgow, UK

**Keywords:** Birthweight, pregnancy outcomes, natural space, sibling study, Scottish Longitudinal Study, Scotland

## Abstract

**Background:**

Birthweight is an important determinant of health across the life course. Maternal exposure to natural space has been linked to higher birthweight, but stronger evidence of a causal link is needed. We use a quasi-experimental sibling study design to investigate if change in the mother’s exposure to natural space between births was related to birthweight, in urban Scotland.

**Methods:**

Amount (% area) of total natural space, total accessible (public) natural space, parks, woodlands and open water within 100 m of the mother’s postcode was calculated for eligible births (*n* = 40 194; 1991–2010) in the Scottish Longitudinal Study (a semi-random 5.3% sample of the Scottish population). Associations between natural space and birthweight were estimated, using ordinary least squares and fixed effects models.

**Results:**

Birthweight was associated with the total amount of natural space around the mother’s home (+8.2 g for interquartile range increase), but was unrelated to specific types of natural space. This whole-sample relationship disappeared in the sibling analysis, indicating residual confounding. The sibling models showed effects for total natural space with births to women who already had children (+20.1 g), and to those with an intermediate level of education (+14.1 g).

**Conclusions:**

The importance of total natural space for birthweight suggests that benefits can be experienced near to as well as within natural space. Ensuring expectant mothers have good access to high quality neighbourhood natural space has the potential to improve the infant’s start in life, and consequently their health trajectory over the life course.

## Introduction

Fetal growth has implications for health across the life course. Low birthweight, for example, has been associated with increased risk of developmental setbacks in childhood,^1^^,^^2^ and illnesses such as type 2 diabetes and cardiovascular disease in adulthood.^3^^,^^4^ Increasing the proportion of babies born with a healthy birthweight has been identified as a priority in many countries, including Scotland.^5^ Individual-level factors are key determinants of fetal growth conditions, but neighbourhood environments are also thought to play a critical role.

Mothers living in more disadvantaged areas are at higher risk for low birthweight births, after accounting for individual characteristics.^6^ Aspects of the social environment such as crime rates and social contacts are strongly related to birthweight, suggesting the importance of mechanisms related to stress and coping.^7^ Increased risk of low birthweight has also been linked to physical environmental factors including noise,^8^ air pollution,^9^ traffic exposures^10^ and the natural environment or ‘green space’.^11^

Evidence for a link between the natural environment and birthweight has emerged over the past 5 years.^12–21^ The evidence to date is entirely from cross-sectional study designs, which are subject to residual confounding (e.g. by inadequately captured socioeconomic position or residential preferences), and do not permit causal inference. Most studies have used a satellite-derived measure of ‘greenness’ surrounding the mother’s residential location, and most of these found a population-wide relationship between residence in greener areas and higher birthweight births,^13–16^^,^^18–21^ although two did not.^12^^,^^17^

Satellite-derived greenness measures, however, cannot differentiate between public and private spaces. The distinction is an important one, because whether the space can be physically entered or just experienced from outside could influence its potential health benefits. The mechanisms by which natural space may influence birth outcomes have been summarized by Kihal-Talantikite *et al*.^22^ as psychosocial (via reduced maternal stress), physiological (via improved maternal health) and environmental (via reduced maternal exposure to environmental risk factors). For example, reduced air and noise pollution might be experienced by a mother living in the vicinity of a natural space, but additional benefits might be accrued if she was able to enter the space and use it for relaxation and/or physical activity. One study found that proximity to public green space was related to population-wide higher mean birthweight,^15^ although others found no relationship.^12^^,^^14^^,^^20^^,^^21^

We used a robust study design to investigate whether the amount of natural space around the homes of mothers in urban Scotland was related to birthweight. Our study is the first to assess relationships with specific types of publicly accessible natural space: parks, woodlands and open water. Each of these natural space types has been linked to improved physiological and psychological health of adults.^23–25^ We then explored variation in the relationship by parity, because we hypothesized that during her pregnancy a woman may spend more time in her local neighbourhood, and make more visits to natural spaces like parks, if she already had children,^26^ and therefore would have increased opportunity for experiencing potential benefits of neighbourhood natural space. Finally, we investigated whether the availability of local green space might offer an opportunity to mitigate health inequalities, by examining if natural space was more strongly related to birthweight for mothers with low educational attainment, as found in some studies.^12^^,^^21^ We used a sibling study design (‘case-crossover’, or within-mother) in which one sibling represented the control case and another was the outcome of the treatment (i.e. a change in the mother’s exposure to natural space).

## Methods

### Study population

Our sample was extracted from the Scottish Longitudinal Study (SLS): National Records Scotland (NRS) records and linked administrative data for a semi-random 5.3% sample of the Scottish population.^27^ The SLS team produced an extract of live births between May 1991 and December 2010 to SLS mothers 16 years of age and over. Some cleaning of this extract was required to produce 48 556 records for linking to National Health Service (NHS) SMR02 records. A sample of singleton births were further extracted after the linking exercise, resulting in a linked file of 46 093 (94.9%). The research was approved by the University of Edinburgh’s Research Ethics and Integrity Committee.

### Natural space exposure

We calculated the amount of natural space around the mother’s home address at the registration of each birth, using Scotland’s Greenspace Map (SGM).^28^ The SGM study area covered settlements in Scotland with populations greater than 3000 (in 2001). Each polygon of a high-resolution (centimetre accuracy) vector map product (Ordnance Survey’s MasterMap) had been manually classified into types (e.g. park, playing field, church yard, private garden, or school ground) using aerial photography from 2009. Our cleaning of the dataset involved removing overlaps and unmapped portions, and adding in agricultural and open water areas from other map products (described in detail in Richardson *et al.* 2017^29^).

‘Total natural space’ included all public and private natural surfaces—vegetation, water, sand, mud and rock—and included private gardens. We defined natural space as ‘accessible’ if it could be accessed by the general public free of charge, which in Scotland includes parks, woodlands, playing fields, play spaces, amenity spaces, golf courses, institutional grounds, cemeteries and churchyards, open semi-natural, agricultural land and open water. Open water was considered accessible because, even if the water is not typically entered on a visit, the adjoining beaches or paths are used for the purpose of visiting the water body. Non-accessible natural spaces included school grounds, bowling greens, allotments, ‘other sports’ grounds (e.g. stadia), and private gardens.

For every postcode in the study area (each representing approximately 15 households) we calculated the proportion of the area of each natural space type (total, accessible, parks, woods and open water) within 100 m, using the GIS software ArcMap 10.1 (ESRI, Redlands, CA). A 100-m buffer is most commonly used in similar studies.^30^ SLS staff linked the natural space measures to each birth to mothers residing within the SGM study area at the registration of the birth (40 194 births to 25 406 mothers).

### Covariates

We adjusted our models for mother, infant and neighbourhood characteristics known to be related to birthweight. Covariates for the infant were sex, parity (nulliparous or multiparous), estimated gestational age (weeks, derived from estimated date of last menstrual period), year of birth and season of conception (based on estimated gestation age: March–May = spring; June–August = summer; September–November = autumn; December–February = winter). As a sensitivity test we categorized gestational age (< 37 weeks, 37–38 weeks, 39–40 weeks, and > 40 weeks), but this made no substantive differences to the results. Covariates for the mother at the time of the birth were age group (16 to 19, 20 to 24, 25 to 29, 30 to 34, 35 to 39 and 40+ years), height (cm), highest educational attainment (no qualifications, school qualifications or post-school qualifications, from the latest census data available), ethnicity (White or non-White), tenure (home owner, social renter, private renter or living rent free, from the nearest census) and whether she smoked while pregnant (yes or no).

We also adjusted for equivalized household wage (in 2006 pounds sterling), estimated from the mother’s and father’s occupations and ages at the registration of the birth.^31^ Household wage was divided by 1.5 for two-parent households (joint/married birth registrations), as per the OECD household equivalence scale.^32^ Neighbourhood-level disadvantage was measured using national-level quintiles of the income deprivation domain of the Scottish Index of Multiple Deprivation (SIMD),^33^ for the mother’s residential ‘datazone’ (administrative unit containing 500–1000 residents). Based on the data collection window for each SIMD version, we used SIMD 2004 for 1991–2003 births, SIMD 2006 for 2004–06 births, SIMD 2009 for 2007–09 births and SIMD 2012 for 2010 births. Income deprivation data were unavailable for the 1990s; we tested the sensitivity of the results to excluding births before 2001 and found no substantive differences. Ambient pollution is a risk factor for lower-weight births,^9^ and hence we obtained annual average estimated concentrations of particulate matter less than 10 µm in diameter (PM_10_) for the 1-km^2^ grid cell in which the mother lived, for the year of the birth [earliest data (1994) used for 1991–94 births, and latest data (2008) used for 2008–10 births].^34^ Missingness rates were highest for whether the mother had smoked during pregnancy (14.7%). To improve the representativeness of the models, 10 sets of imputed variables were generated using multiple imputation in Stata SE/14.0 (StataCorp, College Station, TX).

### Statistical analyses

For comparability with previous work, we first used a standard ordinary least squares (OLS) model to assess the association between maternal exposure to natural space (by type) and birthweight, controlling for observed characteristics. Clustering within mothers was taken into account by estimating robust standard errors. Second, we ran a fixed-effects ‘within-mothers’ model that additionally controlled for all unobserved characteristics that were shared between siblings, as well as capturing the between-mothers effects of the OLS model. An OLS result is considered largely unaffected by residual confounding if it is similar to that from a fixed effects model. The strongest support for a causal influence of natural space on birthweight is from a fixed effects model. After running whole-sample models, we stratified by parity and by the mother’s highest educational attainment. Interaction models were then used as a formal interaction test. Within-mothers models could not be run for first-time mothers because first births only occurred once per mother. We checked for non-linearity of the relationship between natural space and birthweight using categorical natural space variables, but found no evidence that linear models were not appropriate. Analyses were conducted within the SLS safe setting in Edinburgh, UK.

## Results

Descriptive statistics for the variables are given in Tables 1, 2 and 3. Total natural space within 100 m of the mother’s home was positively correlated with accessible space (r = 0.44), parks (r = 0.13), woods (r = 0.19) and open water (r = 0.04; all *P* ≤ 0.0001). Mean birthweight was higher for births to mothers with a greater proportion of total natural space, woodland and open water within 100 m of their homes (Table 3).

**Table 1:** Descriptive statistics for the continuous infant, mother and neighbourhood variables for the 40 194 singleton live births between 1991 and 2010, before imputation

Continuous variable	Missing (%)	Mean (95% CI)	Median (IQR)
Infant:			
Birthweight (g)	17 (< 0.1)	3385.6 (3380.0 to 3391.3)	3410.0 (700.0)
Gestational age (weeks)	27 (0.1)	39.3 (39.3 to 39.3)	40.0 (2.0)
Mother:			
Equivalized household wage (weekly)	0 (0.0)	197.8 (196.7 to 198.9)	182.0 (155.0)
Height (cm)	4268 (11.9)	162.2 (162.1 to 162.2)	162.0 (9.0)
Neighbourhood:			
PM_10_ (µg.m^−3^)	1325 (3.3)	13.7 (13.7 to 13.7)	13.6 (3.9)
Total natural space (% in 100 m)	0 (0.0)	56.8 (56.6 to 56.9)	59.7 (18.9)
Accessible natural space (% in 100 m)	0 (0.0)	16.4 (16.2 to 16.5)	12.4 (20.4)
Parks (% in 100 m)	0 (0.0)	1.7 (1.6 to 1.7)	0.0 (0.0)
Woodland (% in 100 m)	0 (0.0)	1.1 (1.0 to 1.1)	0.0 (0.0)
Open water (% in 100 m)	0 (0.0)	0.6 (0.6 to 0.6)	0.0 (0.0)

Source: SLS.

**Table 2:** Descriptive statistics for the categorical variables for the 40 194 singleton live births, before imputation

Infant variable	Level	Count	% sample	Mother or neighbourhood variable	Level	Count	% sample
Sex	Boy	20686	51.5	Age group	16 to 19	3448	8.6
	Girl	19508	48.5		20 to 24	8104	20.2
Parity	Nulliparous	17616	43.8		25 to 29	11785	29.3
	Multiparous	22578	56.2		30 to 34	10974	27.3
Season of conception	Spring	9347	23.3		35 to 39	4991	12.4
	Summer	9867	24.6		40+	892	2.2
	Autumn	10486	26.1	Ethnicity	White	37035	92.1
	Winter	10467	26.0		Non-White	853	2.1
	Missing	27	0.1		Missing	2306	5.7
Year of birth	1991	1651	4.1	Highest educational attainment	No qualifications	5847	14.6
	1992	2323	5.8		Lower school qualifications	11838	29.5
	1993	2185	5.4		Higher school qualifications	5897	14.7
	1994	2124	5.3		Post-school vocational qualification	5136	12.8
	1995	2047	5.1		Degree/equivalent	10110	25.2
	1996	2047	5.1		Missing	1366	3.4
	1997	2016	5.0	Smoked during pregnancy	No	24662	61.4
	1998	1909	4.8		Yes	9612	23.9
	1999	1955	4.9		Missing	5920	14.7
	2000	1894	4.7	Tenure	Home owner	21156	52.6
	2001	2016	5.0		Social renter	11920	29.7
	2002	1957	4.9		Private renter	2546	6.3
	2003	2021	5.0		Lives rent free	615	1.5
	2004	2078	5.2		Missing	3957	9.8
	2005	1931	4.8	Neighbourhood SIMD Income deprivation quintile	1 (most deprived)	12213	30.4
	2006	2003	5.0		2	8813	21.9
	2007	2100	5.2		3	6597	16.4
	2008	2055	5.1		4	5538	13.8
	2009	1995	5.0		5 (least deprived)	7033	17.5
	2010	1887	4.7				

Source: SLS.

**Table 3:** Mean natural space availability and birthweight for the 40 194 singleton live births between 1991 and 2010, by natural space quantile

Natural space type	Quantile	*n* births	Mean % natural space within 100 m of mother’s home	Mean birthweight (g)
Total natural space	1 (least)	10050	35.0	3341
	2	10048	54.6	3382
	3	10048	63.5	3407
	4 (most)	10048	74.1	3412
Accessible natural space	1 (least)	10050	1.4	3408
	2	10047	8.1	3380
	3	10049	18.0	3383
	4 (most)	10048	38.1	3372
Parks	1 (least)	34104	0.0	3387
	2 (most)	6090	11.0	3379
Woodlands	1 (least)	34776	0.0	3383
	2 (most)	5418	7.9	3401
Open water	1 (least)	35735	0.0	3383
	2 (most)	4459	5.4	3409

Source: SLS.

An interquartile range (IQR) increase in total natural space (the difference between living at the 75th compared with the 25th percentile) was associated with an increase in birthweight in the between-mothers model (+8.2 g; *P* = 0.005), but not in the within-mothers model (Table 4a). Specific types of natural space were not related to birthweight in either model. Table 4b also emphasizes the strong and well-established associations of income, pollution, education and particularly smoking with birthweight.

**Table 4:** Mean change in birthweight (g) for (i) an IQR increase^a^ in natural space amount (by type) within 100 m of mother’s home, and (ii) selected covariates (from the total natural space model); 95% confidence intervals shown in parentheses

	Between mothers	Within mothers
(i)		
Total natural space	8.19 (2.43 to 13.95)**	6.36 (−2.68 to 15.40)
Accessible natural space	0.11 (−6.20 to 6.42)	3.42 (−5.41 to 12.24)
Parks	−0.57 (−1.32 to 0.18)	−0.38 (−0.87 to 0.10)
Woodlands	−0.27 (−1.37 to 0.83)	1.46 (−0.21 to 3.13)
Open water	0.46 (−1.04 to 1.95)	1.04 (−1.24 to 3.32)
(ii)		
Smoker (ref: non-smoker)	−216.14 (−228.79 to − 203.49)***	−83.69 (−102.71 to − 64.66)***
Equivalized household wage (per £100/week)	16.97 (10.45 to 23.50)***	9.40 (−0.87 to 19.67)$
Highest educational attainment (ref: no qualifications)		
Lower school	24.38 (7.69 to 41.07)**	
Higher school	31.41 (11.91 to 50.91)**	
Post-school vocational	34.56 (14.15 to 54.96)**	
Degree	29.49 (9.95 to 49.02)**	
PM_10_ (per 10 µg/m^3^)	−40.62 (−69.84 to − 11.41)**	30.42 (−25.67 to 86.50)

Source: SLS.

a IQRs for between-mother change in natural space given in Table 1. IQRs for within-mother change in natural space are: total 18.5 percentage points, accessible 18.6, parks 0.4, woodlands 0.0 and open water 0.0. Results given for 1 percentage point increase where IQR = 0.

** 0.001 ≤ *P*< 0.01;

*** *P* < 0.001.

Total natural space within 100 m of home was not related to birthweight for first-time mothers, but an IQR increase was related to a + 12.9 g increase in birthweight (*P* = 0.002) for mothers who already had children (Table 5a). The effect size increased in the within-mothers models (+20.1 g, *P* = 0.013) (within-mothers models could not be run for first-time mothers). Parity interacted in the relationship between total natural space and birthweight: the interaction term for mothers with previous children for an IQR increase in total natural space was +17.5 g (*P* = 0.002).

**Table 5:** Mean change in birthweight (g) for an IQR increase^a^ in natural space amount (by type) within 100 m of mother’s home, by (i) parity, or (ii) highest educational attainment; 95% confidence intervals shown in parentheses

		(a) Parity		(b) Highest educational attainment		
		No previous children^b^	Has previous children	No qualifications	School	Degree/equivalent
*n* births		17616	22578	6214	18440	15495
*n* mothers		17616	17049	3806	11574	9972
Between-mothers	Total natural space	2.86 (−4.75 to 10.46)	12.87 (4.92 to 20.82)**	7.17 (−6.80 to 21.15)	9.83 (1.25 to 18.41)*	6.15 (−3.79 to 16.08)
	Accessible natural space	0.50 (−7.89 to 8.89)	−0.07 (−8.52 to 8.37)	4.75 (−11.70 to 21.20)	1.14 (−7.98 to 10.27)	−3.59 (−14.20 to 7.01)
	Parks	−0.99 (−2.03 to 0.06)	−0.25 (−1.24 to 0.74)	−0.34 (−2.29 to 1.61)	−0.86 (−1.94 to 0.22)	−0.30 (−1.55 to 0.95)
	Woodlands	−0.28 (−1.65 to 1.08)	−0.30 (−1.78 to 1.18)	0.69 (−2.35 to 3.72)	−0.30 (−2.01 to 1.40)	−0.53 (−2.19 to 1.14)
	Open water	0.52 (−1.32 to 2.35)	0.46 (−1.73 to 2.64)	4.75 (−0.65 to 10.14)	−0.54 (−2.61 to 1.53)	0.32 (−1.94 to 2.57)
Within-mothers	Total natural space		20.12 (4.32 to 35.92)*	0.70 (−20.40 to 21.79)	14.07 (0.82 to 27.31)*	−1.19 (−17.53 to 15.15)
	Accessible natural space		11.11 (−4.09 to 26.32)	11.23 (−9.68 to 32.15)	2.88 (−10.06 to 15.81)	0.12 (−15.55 to 15.80)
	Parks		0.28 (−0.55 to 1.10)	−0.09 (−1.17 to 0.99)	−0.53 (−1.26 to 0.19)	−0.47 (−1.35 to 0.40)
	Woodlands		1.57 (−1.30 to 4.44)	4.40 (−0.28 to 9.07)	0.86 (−1.67 to 3.39)	1.34 (−1.45 to 4.14)
	Open water		−1.03 (−5.00 to 2.94)	0.31 (−7.16 to 7.78)	0.94 (−2.44 to 4.31)	1.37 (−2.04 to 4.78)

Source: SLS.

a IQRs for between-mother change in natural space given in Table 1. IQRs for within-mother change in natural space are: total 18.5 percentage points, accessible 18.6, parks 0.4, woodlands 0.0 and open water 0.0. Results given for 1 percentage point increase where IQR = 0.

b Within-mothers models could not be run for mothers with no previous children.

** 0.001 ≤ P < 0.01;

* 0.01 ≤ *P* < 0.05.

Natural space was related to birthweight for mothers with school-level education, but not those with no qualifications or degree-level qualifications (Table 5b). An IQR increase in total natural space within 100 m was associated with a + 9.8 g (*P* = 0.025) between-mothers increase in birthweight for mothers with school qualifications, which increased to + 14.1 g in the within-mothers model (*P* = 0.044). We found no interaction effect, however.

## Discussion

In a nationally representative sample of mothers in urban Scotland we found that having more natural space surrounding the maternal home was associated with higher birthweight. Importantly, the sibling analysis showed robust relationships for mothers who already had children (although the models could not be run for first-time mothers), and mothers whose highest educational qualifications were school level (rather than no qualifications or higher qualifications). No independent relationships with birthweight were found for individual natural space types.

Most previous studies have found that mothers residing in areas with higher greenness or more green space have higher birthweight births.^13–16^^,^^18–21^ We found a modest population-wide between-mothers effect size of + 8.2 g for an IQR increase in natural space area within 100 m, although others considering the same buffer size (and controlling for similar covariates) found either no effect ^12^^,^^18^^,^^20^ or effect sizes of + 15.8 to + 22.8 g for an IQR increase in greenness.^14^^,^^15^^,^^21^ Our whole-population finding was subject to residual confounding, as it disappeared in the sibling analysis. Ours is the first sibling design study of birthweight and natural space, so it is possible that the whole-population results from other studies might also have been subject to confounding from unobserved characteristics.

We hypothesized that particular types of public (accessible) natural space might be more strongly related to birthweight than total natural space, as the latter combines public and private (typically unusable) spaces. However, we found no relationship for birthweight with total accessible space, parks, woodlands or open water. Five other studies have assessed birthweight relationships both with total green space (or greenness) and with public space availability: one found links for both types,^15^ another found links for neither^20^ and most found links for total but not public green space.^12^^,^^14^^,^^21^ Taken together, this body of evidence suggests that ambient neighbourhood naturalness may be more influential for healthy fetal growth than public natural space. This points to potential causative pathways that can be experienced near natural spaces as well as within them, such as stress reduction or amelioration of environmental risk factors.^35^

The OLS models showed that total natural space was related to birthweight for births to women who already had children, but not for first-borns. The sibling analysis confirmed the effect for mothers with children: an effect that was larger than that for a £100 increase in weekly wage, and was comparable to the effect of a lower school education versus no education. The sibling analysis was not possible for first-time mothers, but given the small OLS coefficient for first-time mothers and high *P*-value (*P* = 0.46), it is unlikely that additional control for unobserved explanatory factors would have revealed a relationship. Child care responsibilities may lead women with children to spend more time in their neighbourhood and increase their exposure to local environments.^26^ Women with children are less likely to exercise regularly than women without children,^36^ but they are 25% more likely to mention using a natural area (e.g. park or woodland) for some form of physical activity, according to weighted tabulation of 2014 Scottish Health Survey data [nesstar.ukdataservice.co.uk]. This suggests that the beneficial relationship with birthweight we found for mothers who already had at least one child is more likely due to the restorative experiences or positive social connections facilitated by their natural space visits with their children, rather than from physical activity per se. Concomitant with the health benefits conferred on an infant via higher birthweight, mothers who use neighbourhood natural space more frequently also raise children who are more likely to use and benefit from such spaces during childhood and into adulthood.^29^^,^^37^ Urban planning that ensures high quality and equitable availability of neighbourhood natural space for expectant mothers and families has the potential to improve health across the life course.

A growing body of research suggests that green or natural space can buffer the detrimental influences of social disadvantage on various health outcomes,^38^^,^^39^ including the educational attainment gradient in birth outcomes.^12^^,^^21^ We therefore hypothesized that the relationship between natural space and birthweight would be strongest for the least-educated mothers; instead, we only found a relationship for mothers with an intermediate level of education (school-level qualifications). This finding is consistent with the only other UK study of natural space and birth outcomes,^14^ which found the relationship to be restricted to mothers with school qualifications, rather than for those with less education. We know that education can enhance opportunities for employment, higher earnings, safer homes and access to healthful resources, and whereas we have provided good evidence that natural space has benefits for birthweight, it is possible that these benefits cannot outweigh the multitude of disadvantageous circumstances that often afflict the least educated mothers. Further work would be needed to ascertain why this finding might be specific to the UK context. At the other end of the educational spectrum, mothers with the highest qualifications may also not experience benefits from neighbourhood natural space because they have higher mobility and may spend less time in their residential neighbourhood.^40^

Our study has strengths that make it a substantial contribution to the existing literature. We exploited a large population database of linked administrative and health data, and were able to link in detailed natural space information from the high quality and detailed SGM dataset. The large sample gave our models substantial statistical power, and enabled us to adjust for many relevant confounders at the infant, mother, household and area levels. Our principal contribution to this field is the use of a sibling study for the first time, enabling us to produce the clearest evidence to date of a causal link between natural space and birthweight.

Some limitations must also be acknowledged. First, the natural space and area deprivation measures were linked to each birth based on the mother’s address at the registration of the birth, but this address may have changed during the pregnancy. There may be a degree of exposure misclassification for some births therefore, although previous longitudinal studies have shown that people tend to move between very similar types of physical environment.^41^ Second, the natural space data refer to a single point in time (2009), whereas the births ranged from 1991 to 2010. Comparing the 2009 SGM data with a 1969 dataset for Edinburgh, however, showed only minimal change in the distribution of natural spaces over this 40-year period,^42^ suggesting that our measure is applicable back to 1991. Third, we have only measured the amount of residential surrounding natural space, but we do not know how each mother experienced the space around her home. How the natural space is interacted with will affect potential health benefits. Fourth, a mother’s pre-pregnancy weight status is related to birthweight, but these data were unavailable for our sample.

## Conclusions

Birthweight is an important determinant of subsequent health across the life course, and in Scotland has been identified as one of the country’s National Indicators for monitoring the progress of government.^5^ We found that birthweight is associated with the total amount of natural space around the mother’s home, regardless of whether the space is public or private. The importance of total natural space rather than public space suggests potential causative mechanisms that can operate near natural spaces as well as within them, such as stress reduction or the amelioration of environmental risk factors.

Sibling analysis showed that the relationship was most robust for mothers who already had children, and mothers whose highest educational qualifications were school-level. Ensuring good, equitable availability of neighbourhood natural space for expectant mothers, and enabling particularly those expecting their first child to interact with natural space, has the potential to improve the infant’s start in life and consequently their health trajectory over the life course.

## Funding

This work was supported by the European Research Council [ERC-2010-StG Grant 263501]. RM was funded by the UK Medical Research Council as part of the Neighbourhoods and Communities Programme (MC_UU_12017-10). The LSCS is supported by the ESRC/JISC, the Scottish Funding Council, the Chief Scientist’s Office and the Scottish Government. The authors alone are responsible for the interpretation of the data. 
